# Does Ethnicity Impact Outcome Following Cardiac Surgery?

**DOI:** 10.1186/1749-8090-10-S1-A157

**Published:** 2015-12-16

**Authors:** Pankaj Kumar Mishra, Andrew Panayiotou, Alan Nevill, Matthew Thomas, Heyman Luckraz

**Affiliations:** 1 Department of Cardiothoracic Surgery, Heart and Lung Centre, Wolverhampton, UK; 2 New Cross Hospital, Wolverhampton, UK; 3 Research Institute of Healthcare Sciences, University of Wolverhampton, Wolverhampton, UK

## Background/Introduction

Impact of ethnicity on outcome following cardiac surgery is controversial. Current risk stratification models do not include ethnicity as risk factor.

## Aims/Objectives

To assess impact of ethnicity (South-East Asian versus Caucasian) on outcome following cardiac surgery

## Method

Patients of Asian ethnicity who underwent cardiac surgery at our unit between Sep 2005 to Dec 2013 were included in the study (n = 855). This group was matched 1:2 with Caucasian patients (n = 1710).

## Results

Pre-operative characteristics confirmed that patients of Asian ethnicity were more likely to be younger [Mean Age 61.7 (SD 11.3) v/s 63.2 (SD 10.4) years, p =0.006], females (23% v/s 19%, p = 0.03) with lower BMI (27 vs 29, p < 0.01) as compared to Caucasian population. Asian ethnicity was strongly associated with higher prevalence of diabetes (51% v/s 22%, p = 0.01), non-smokers (69% v/s 30%, p =< 0.01) and need for urgent surgery (42% v/s 29%, p < 0.01). Post operatively patients with Asian Ethnicity had a higher re-exploration rate (7.4% v/s 5.4%, p < 0.04), higher rate of readmission to ITU (2.9% v/s 2.6%, p = 0.04), a greater need for blood and blood products transfusion requirement (Blood Units 1.23 v/s 0.76, p < 0.01) and was also associated with a higher in-hospital mortality (2.8% v/s 1.5%, p = 0.02). Caucasians had a significantly higher prevalence of post op AF (26% v/s 17%, p < 0.01) but shorter ITU (median 1.0 (0,90) days v/s. 1.5 (0,183) days, p < 0.01) and in-hospital stay (median 5 (1,184) days v/s. 6 (3,183), p < 0.01).

## Discussion/Conclusion

Asian ethnicity has an adverse impact on outcome following cardiac surgery, in a matched population. If confirmed in large randomised studies, ethnicity should be made part of future risk stratification models.

